# Supplementary material for: Median raphe serotonergic neurons projecting to the interpeduncular nucleus control preference and aversion
Source: Nat Commun. 2022 Dec 22;13:7708. doi: 10.1038/s41467-022-35346-7 (PMC9780347; doi:10.1038/s41467-022-35346-7)
Supplement: Supplementary file 1 — Supplementary Information [file 41467_2022_35346_MOESM1_ESM.pdf]

**Supplementary Information**

# Median raphe serotonergic neurons projecting to the interpeduncular nucleus control preference and aversion

Hiroyuki Kawai[1,2,6], Youcef Bouchekioua[3,6], Naoya Nishitani[1,3,4], Kazuhei Niitani[4], Shoma Izumi[4], Hinako Morishita[1], Chihiro Andoh[1], Yuma Nagai[1], Masashi Koda[1], Masako Hagiwara[1], Koji Toda[5], Hisashi Shirakawa[1], Kazuki Nagayasu[1*], Yu Ohmura[3*], Makoto Kondo[2], Katsuyuki Kaneda[4], Mitsuhiro Yoshioka[3] and Shuji Kaneko[1*]

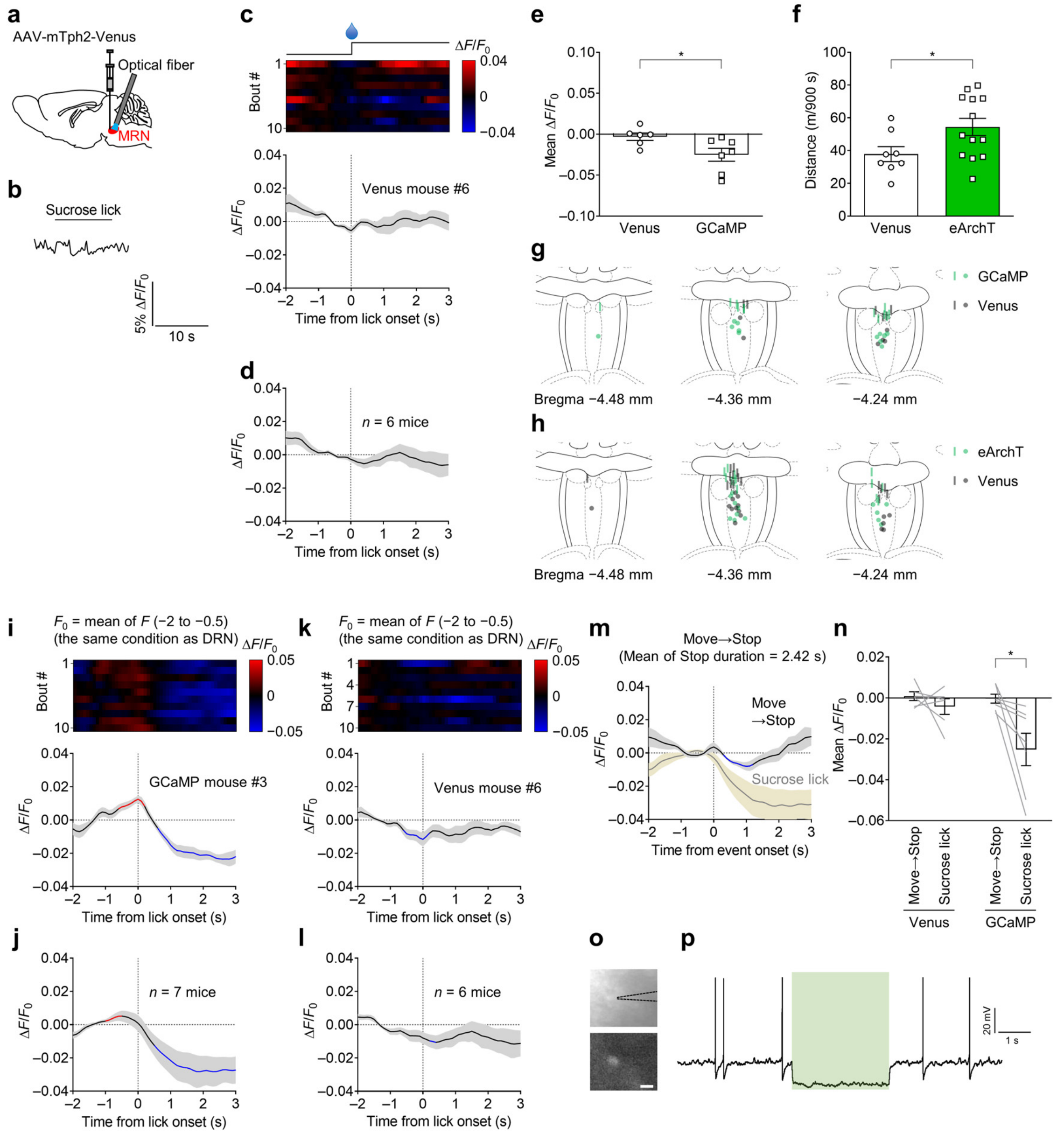


**Supplementary Fig. 1 | Fluorescence change by rewarding stimuli and termination of locomotion and effect of optogenetic inhibition of MRN serotonergic neurons on locomotor activity and activity of MRN serotonergic neurons.**

**a**, Schematic representation of experiments. **b**, Representative raw trace of Venus fluorescence changes before, during, and after sucrose licking. **c**, Top: heatmap of signals (red–blue, high–low). One licking bout per row. Bottom: averaged signals from the MRN in one mouse. $n$ = 10 bouts. **d**, Mean of Venus signals for six mice. **e**, The mean $\Delta F/F_0$ at 3-s after the lick onset (two-tailed unpaired $t$-test (Venus vs GCaMP), $t_{11}$ = 2.317, *$P$ = 0.0408, $n$ = 6 (Venus) and 7 (GCaMP) mice). **f**, Traveled distances in posttest session of

the CPP test (two-tailed unpaired *t*-test (Venus vs eArchT), $t_{19}$ = 2.176, *$P$ = 0.0424, $n$ = 8 (Venus) and 13 (eArchT) mice). **g**, **h**, Verification of virus injection and fiber implantation sites in the MRN for photometry recording (**g**) and optogenetic manipulation (**h**). Green (**g**): GCaMP6s (circle), fiber tip (line), Green (**h**): eArchT3.0 (circle), fiber tip (line), Gray: Venus (circle), fiber tip (line). **i–l**, Fluorescence in the MRN with baseline setting for the DRN. $F_0$ was defined as mean fluorescence from −2 to −0.5 s from licking. **m**, GCaMP fluorescence in the MRN before and after termination of locomotion. **n**, The mean $\Delta F/F_0$ of 3-s after event for the GCaMP mice and Venus mice (two-tailed paired *t*-test (Venus), $t_5$ = 0.8866, $P$ = 0.4159, $n$ = 6 mice, (GCaMP), $t_6$ = 2.935, *$P$ = 0.0261, $n$ = 7 mice). **o**, Representative micrographs of eArchT-eYFP-positive neurons. Top: bright field. Bottom: fluorescence. The dashed lines indicate patch pipettes. Scale bar = 30 μm. **p**, Representative traces of current-clamp recordings from MRN eArchT-eYFP-positive neurons. Green light induced hyperpolarization, which suppressed firing activities induced by a depolarizing current injection. 30 sweeps in 6 cells from 3 mice. The green shaded area represents light stimulation. In **c, d, i-m,** lines and shaded areas indicate mean and s.e.m., respectively. Red and blue segments indicate a statistically significant increase and decrease from the baseline ($P$ < 0.05; permutation test). In other panels, data are presented as mean ± s.e.m. Error bars indicate s.e.m. Source data are provided as a Source Data file.

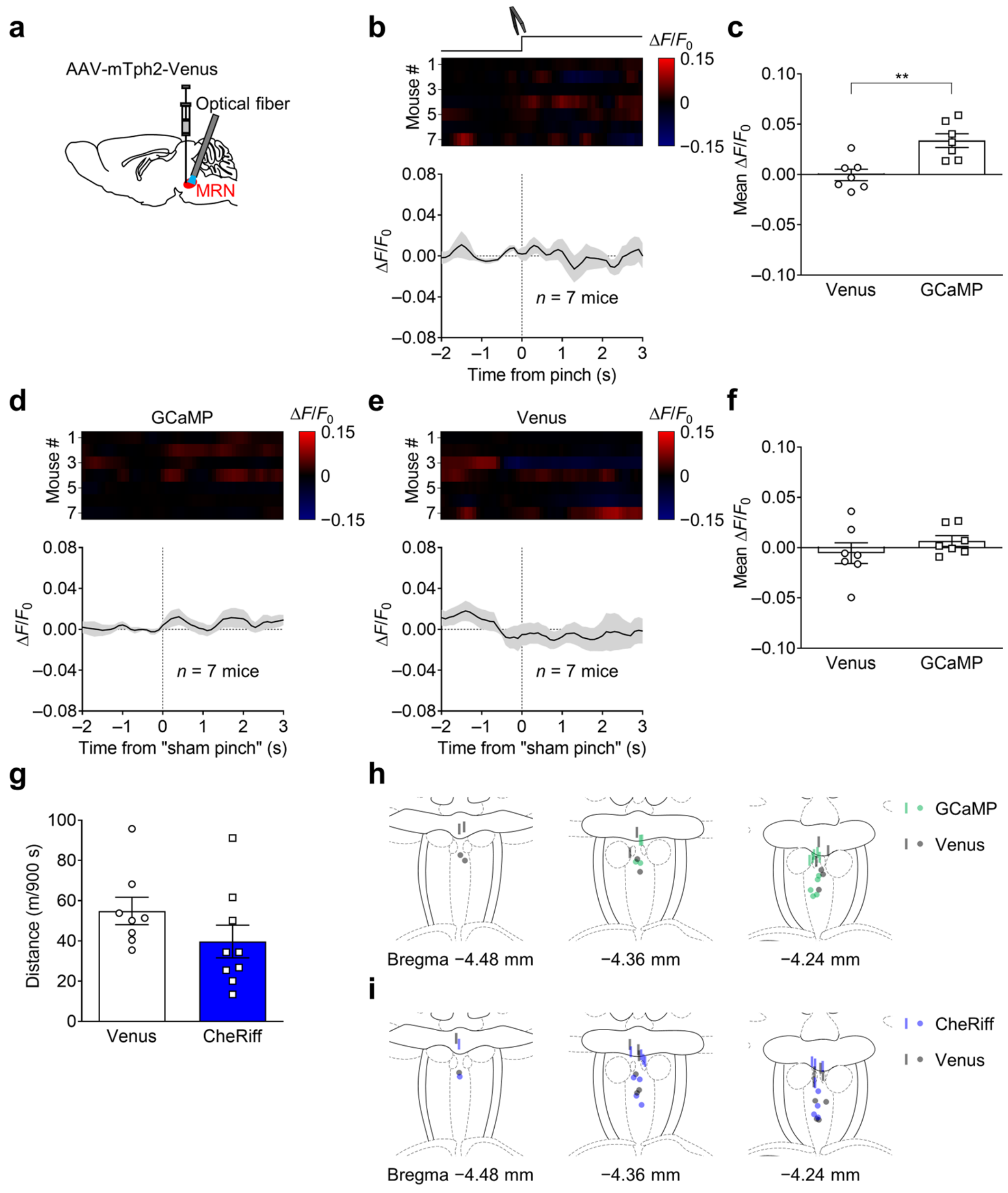


**Supplementary Fig. 2 | Fluorescence change by aversive stimuli and effect of optogenetic activation of MRN serotonergic neurons on locomotor activity.**

**a**, Schematic representation of AAV injection and fiber implantation sites. **b**, Top: heatmap of Venus signals (red–blue, high–low) for each trial (one trial per mouse). Each row represents the data of one mouse. $n$ = 7 mice. Bottom: averaged traces of Venus signals. Lines and shaded areas indicate mean and s.e.m., respectively. **c**, The mean $\Delta F/F_0$ of 3-s after pinch for the GCaMP mice and Venus control mice (two-tailed unpaired $t$-test (Venus vs GCaMP), $t_{12} = 3.851$, $^{**}P = 0.0023$, $n$ = 7 mice per group). **d, e**, Top: heatmap

of GCaMP (**d**) and Venus (**e**) signals (red–blue, high–low) for each "sham pinch" trial (the mouse was approached with the clamp, but the tail was not pinched; one trial per mouse). Each row represents the data of one mouse. $n$ = 7 mice. Bottom: averaged traces of signals. Lines and shaded areas indicate mean and s.e.m., respectively. **f**, The mean $\Delta F/F_0$ of 3-s after "sham pinch" for the GCaMP mice and Venus control mice (two-tailed unpaired $t$-test (Venus vs GCaMP), $t_{12}$ = 1.159, $P$ = 0.2690, $n$ = 7 mice per group). The time when the clamp was closest to the mouse tail was defined as time 0. **g**, Traveled distances in the Venus and CheRiff mice in posttest session of the CPA test (two-tailed unpaired $t$-test (Venus vs CheRiff), $t_{15}$ = 1.416, $P$ = 0.1773, $n$ = 8 (Venus) and 9 (CheRiff) mice). **h**, **i**, Verification of virus injection and fiber implantation sites in the MRN for photometry recording (**h**) and optogenetic manipulation (**i**). Green (**h**): GCaMP6s (circle), fiber tip (line), Blue (**i**): CheRiff (circle), fiber tip (line), Gray: Venus (circle), fiber tip (line). Data are presented as mean ± s.e.m. Error bars indicate s.e.m. Source data are provided as a Source Data file.

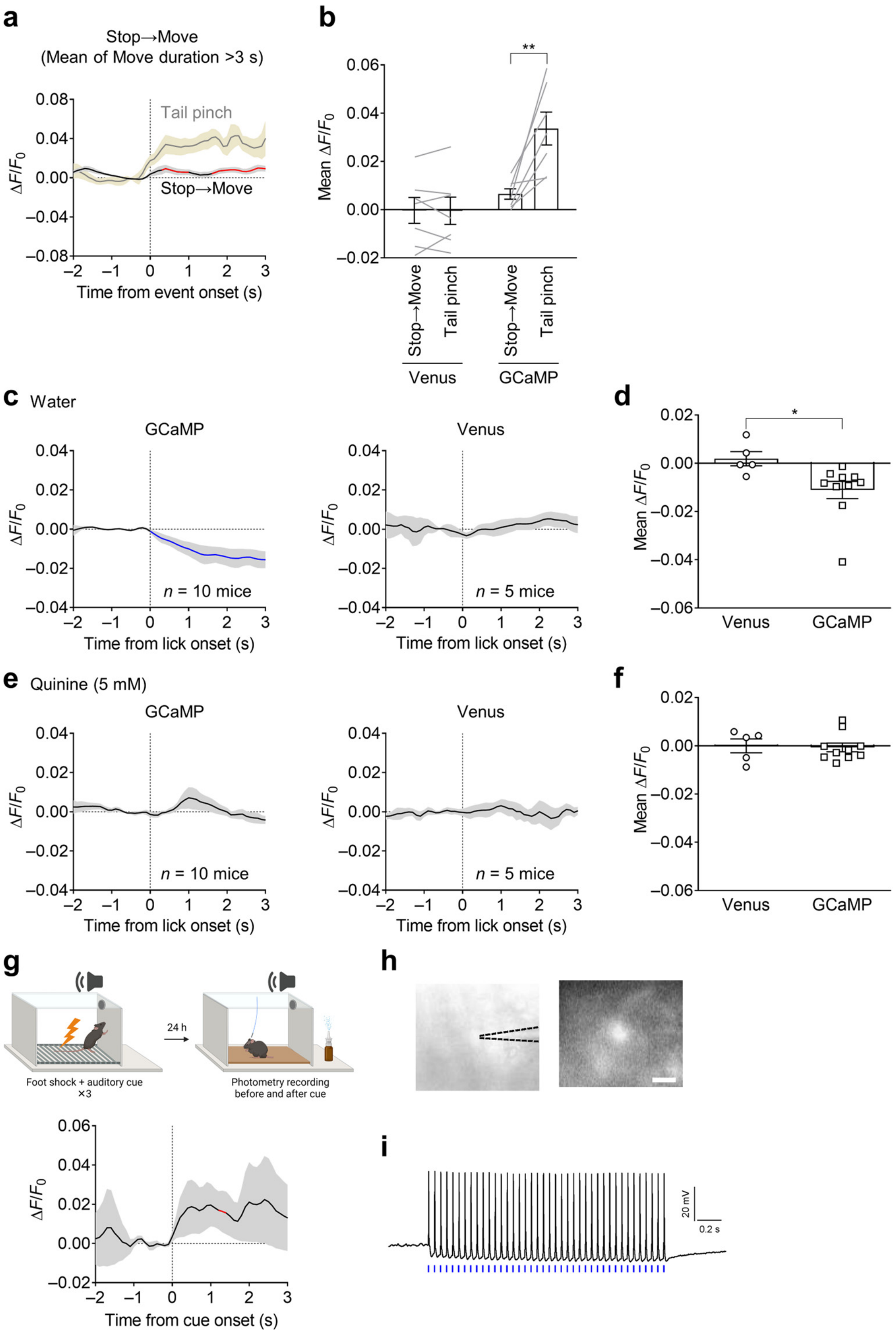


**Supplementary Fig. 3 | Fluorescence change by initiation of locomotion and aversive stimuli and validation of optogenetic activation of MRN serotonergic neurons.**

**a**, Analysis of GCaMP fluorescence in the MRN before and after initiation of locomotion.

Red segments indicate a statistically significant increase from the baseline ($P$ < 0.05; permutation test). **b**, The mean $\Delta F/F_0$ of 3-s after event for the GCaMP mice and Venus mice (two-tailed paired $t$-test (Venus), $t_6$ = 0.06923, $P$ = 0.9471, $n$ = 7 mice, (GCaMP), $t_6$ = 4.127, **$P$ = 0.0062, $n$ = 7 mice). **c–f**, GCaMP and Venus fluorescence in the MRN before and after licking of water (**c**, **d**) or quinine solution (**e**, **f**). Blue segments indicate a statistically significant decrease from the baseline ($P$ < 0.05; permutation test). two-tailed unpaired $t$-test (water), $t_{13}$ = 2.352, *$P$ = 0.0351, $n$ = 5 (Venus) and 10 (GCaMP) mice, (quinine), $t_{13}$ = 0.2024, *$P$ = 0.8428, $n$ = 5 (Venus) and 10 (GCaMP) mice. **g**, GCaMP fluorescence in the MRN before and after auditory cue associated with foot shocks 24 h after conditioning. Red segments indicate a statistically significant increase from the baseline ($P$ < 0.05; permutation test). n = 5 mice. Illustrations were created with BioRender.com. **h**, Representative micrographs of CheRiff-eGFP-positive neurons recorded from MRN slices. Top: bright field image. Bottom: fluorescence image. The dashed lines indicate patch pipettes. Scale bar = 30 μm. **i**, Representative traces of current-clamp recordings from CheRiff-eGFP-positive neurons in the MRN. Blue LED light stimulation (20 Hz frequency, 10 ms duration, 40 pulses) evoked action potentials with 80.4 ± 5.5% fidelity (32.2 ± 2.2 action potentials / 40 pulses, $n$ = 5 cells from 2 mice). The blue lines represent light stimulation. Data are presented as mean ± s.e.m. Error bars indicate s.e.m. Source data are provided as a Source Data file.

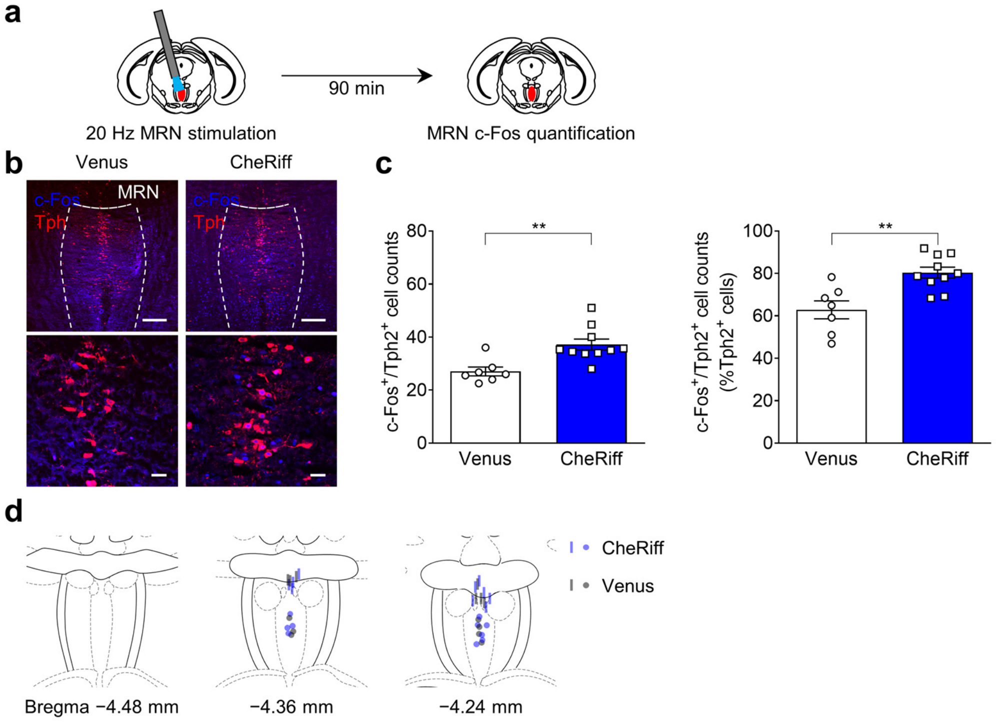


**Supplementary Fig. 4 | c-Fos expression in the MRN after optogenetic activation.**

**a**, Schematic of the experimental design. AAVdj-mTph2-CheRiff-eGFP was injected into the MRN, and an optical fiber was inserted above the MRN. 90-min after MRN stimulation (20 Hz), mice were sacrificed, and c-Fos expression was analyzed. **b**, Representative images of c-Fos-positive (blue) and Tph-positive (red) cells in the MRN. Scale bars indicate 200 µm (top) and 20 µm (bottom). **c**, Quantification of c-Fos-positive cells in the MRN. Left: the number of c-Fos/Tph double-positive cells (two-tailed unpaired *t*-test (Venus vs CheRiff), $t_{15} = 3.588$, $^{**}P = 0.0027$, $n = 7$ (Venus) and 10 (CheRiff) mice). Right: the percentage of c-Fos/Tph double-positive cells relative to Tph-positive cells (two-tailed unpaired *t*-test (Venus vs CheRiff), $t_{15} = 3.758$, $^{**}P = 0.0019$, $n = 7$ (Venus) and 10 (CheRiff) mice). **d**, Verification of virus injection and fiber implantation sites in the MRN. AAV injection and fiber implantation sites were histologically verified under the microscope. Blue circle: AAVdj-mTph2-Cheriff-eGFP, Gray circle: AAVdj-mTph2-Venus-WPRE, Blue line: the tip of fiber for CheRiff mice, Gray line: the tip of the fiber for Venus mice. Data are presented as mean ± s.e.m. Error bars indicate s.e.m. Source data are provided as a Source Data file.

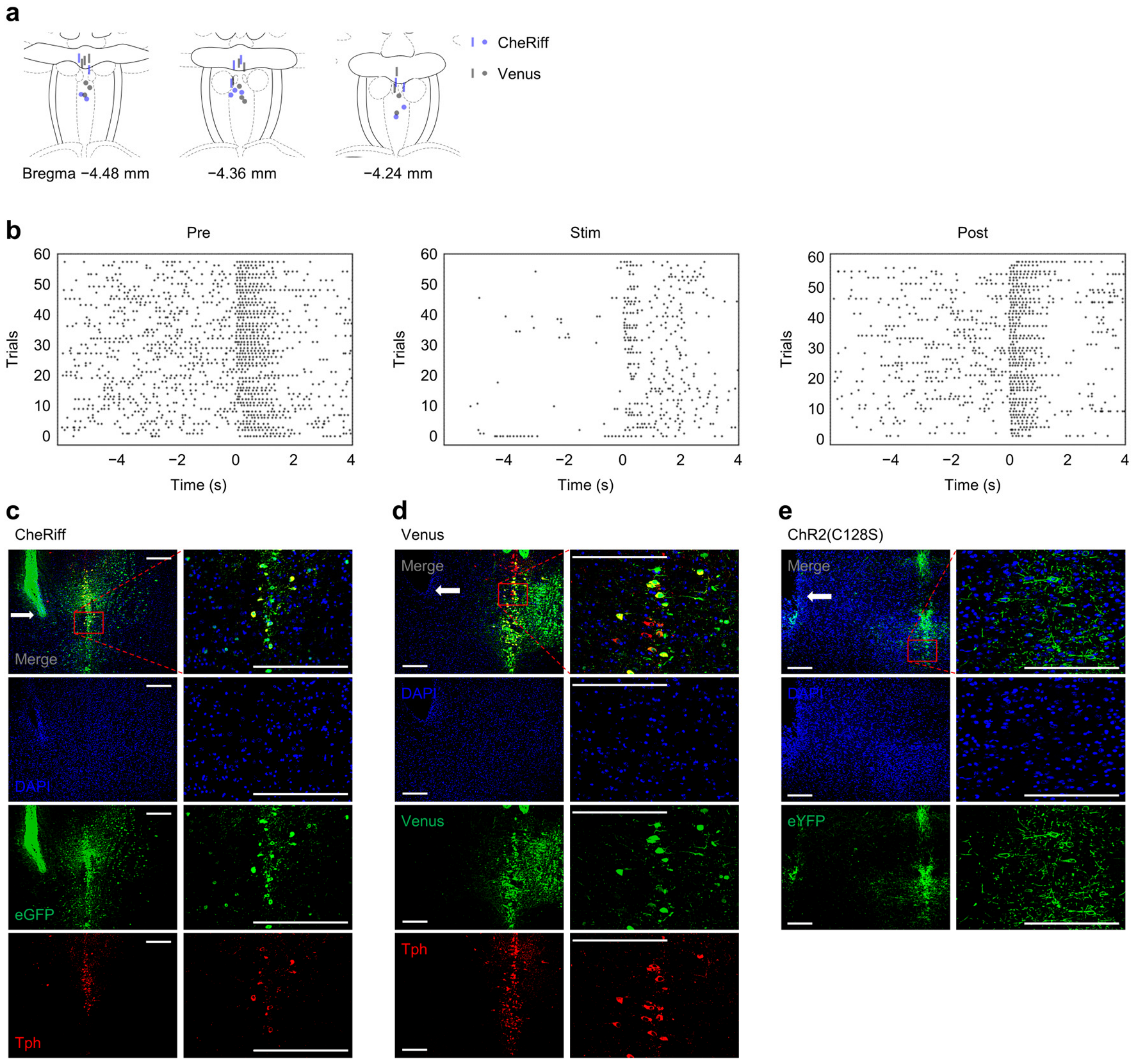


**Supplementary Fig. 5 | Raster plots of licking behavior in the fixed-time schedule task and immunohistochemical verification of transgene expression.**

**a**, Verification of virus injection and fiber implantation sites in the MRN related to Fig. 3 and 4. AAV injection and fiber implantation sites were histologically verified under the microscope. Blue circle: AAVdj-mTph2-CheRiff-eGFP, Gray circle: AAVdj-mTph2-Venus-WPRE, Blue line: the tip of the fiber for CheRiff mice, Gray line: the tip of the fiber for Venus mice. **b**, Representative raster plots during the delivery of a sucrose solution in the fixed-time schedule task for the CheRiff group. Each row represents data from one licking session. The number of licks in each phase (left: Pre; center: Stim; right: Post) was strongly correlated with blue light stimulation, as in Fig. 3h. **c**, Verification of virus injection and fiber implantation sites for the CheRiff mice. After completion of the behavioral analysis, mice were sacrificed, and their brain was collected, frozen and

sectioned. AAV injection and fiber implantation sites were histologically verified under the microscope. Each image represents, in order from top to bottom, merged, DAPI-positive (blue), eGFP-positive (green), and Tph-positive (red) cells. Scale bars indicate 100 µm. The tip of the white arrow indicates the tip of the fiber. **d**, Verification of virus injection and fiber implantation sites for the Venus mice. After completion of the behavioral analysis, mice were sacrificed, and their brain was collected, frozen and sectioned. AAV injection and fiber implantation sites were histologically verified under the microscope. Each image represents, in order from top to bottom, merged, DAPI-positive (blue), Venus-positive (green), and Tph-positive (red) cells. Scale bars indicate 100 µm. The tip of the white arrow indicates the tip of the fiber. **e**, Verification of fiber implantation site for ChR2(C128S) mice. After the completion of behavioral analysis, mice were sacrificed, and the brain was collected, frozen, and sectioned. The AAV injection site and the fiber implantation site were histologically verified under the microscope. Each image represents, in order from top to bottom, merged, DAPI-positive (blue), eYFP-positive (green), and Tph-positive (red) cells. Scale bars indicate 100 µm. The tip of the white arrow indicates the tip of the fiber. Source data are provided as a Source Data file.

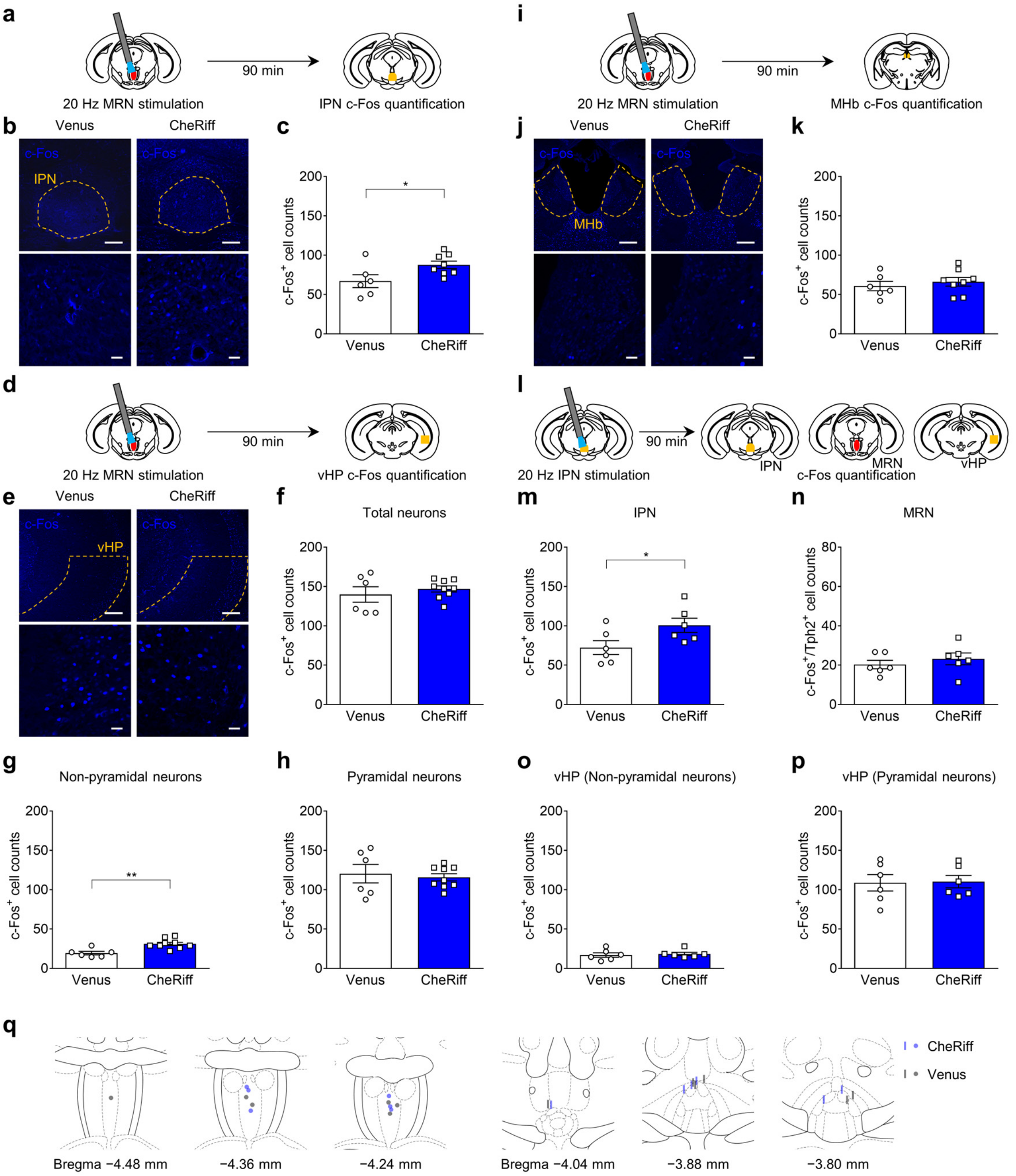


**Supplementary Fig. 6 | c-Fos expression in the IPN, vHP, MHb, and MRN after light stimulation.**

**a, d, i**, Schematic of the experiment. AAVdj-mTph2-CheRiff-eGFP was injected into the MRN. 90-min after MRN stimulation (20 Hz), mice were sacrificed and c-Fos expression in the IPN (**a**), vHP (**d**), and MHb (**i**) were analyzed. **b, e, j**, Representative images for c-Fos-positive (blue) cells in the IPN (**b**), vHP (**e**), and MHb (**j**). Scale bars = 200 (top) and 20 μm (bottom). **c, f–h, k**, Quantification of c-Fos-positive cells in the IPN (**c**), total neurons in vHP (**f**), non-pyramidal neurons in vHP (**g**), pyramidal neurons in vHP (**h**),

and MHb (**k**). IPN: (Venus vs CheRiff), $t_{12}$ = 2.329, *$P$ = 0.0382, $n$ = 6 (Venus) and 8 (CheRiff) mice; vHP (total): (Venus vs CheRiff), $t_{13}$ = 0.7657, $P$ = 0.4575, $n$ = 6 (Venus) and 9 (CheRiff) mice; vHP (non-pyramidal): (Venus vs CheRiff), $t_{13}$ = 3.806, **$P$ = 0.0022, $n$ = 6 (Venus) and 9 (CheRiff) mice; vHP (pyramidal): (Venus vs CheRiff), $t_{13}$ = 0.4284, $P$ = 0.6754, $n$ = 6 (Venus) and 9 (CheRiff) mice; MHb: (Venus vs CheRiff), $t_{12}$ = 0.6630, $P$ = 0.5199, $n$ = 6 (Venus) and 8 (CheRiff) mice. **l,** Schematic of the experiment. AAVdj-mTph2-CheRiff-eGFP was injected into the MRN. 90-min after IPN stimulation (20 Hz), mice were sacrificed and c-Fos expression in the IPN (**m**), MRN (**n**), and vHP (**o, p**) were analyzed. IPN: (Venus vs CheRiff), $t_{10}$ = 2.244, *$P$ = 0.0487; MRN: (Venus vs CheRiff), $t_{10}$ = 0.7810, $P$ = 0.4529; vHP (non-pyramidal): (Venus vs CheRiff), $t_{10}$ = 0.4074, $P$ = 0.6923; vHP (pyramidal): (Venus vs CheRiff), $t_{10}$ = 0.1148, $P$ = 0.9109; $n$ = 6 (Venus) and 6 (CheRiff) mice. **q,** Verification of virus injection and fiber implantation sites in the MRN (left) and IPN (right). AAV injection and fiber implantation sites were histologically verified under the microscope. Blue: Cheriff (circle), fiber tip (line), Gray: Venus (circle), fiber tip (line). Two-tailed unpaired $t$-test was used for statistical analyses. Data are presented as mean ± s.e.m. Error bars indicate s.e.m. Source data are provided as a Source Data file.

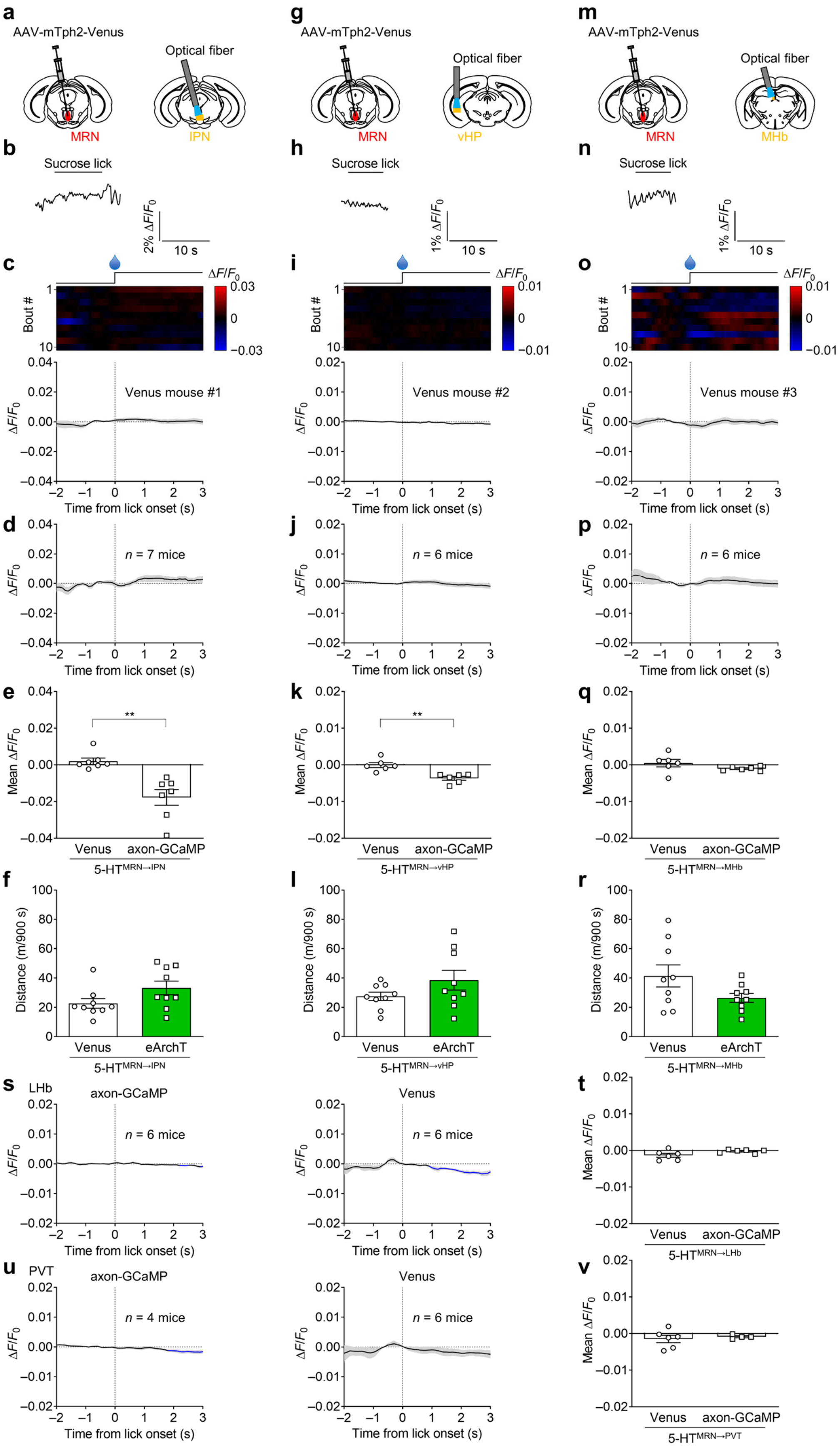
a
AAV-mTph2-Venus
Optical fiber
MRN
IPN
g
AAV-mTph2-Venus
Optical fiber
MRN
vHP
m
AAV-mTph2-Venus
Optical fiber
MRN
MHb
b
Sucrose lick
2% ΔF/F0
10 s
h
Sucrose lick
1% ΔF/F0
10 s
n
Sucrose lick
1% ΔF/F0
10 s
c
i
o
Bout #
ΔF/F0
Venus mouse #1
Venus mouse #2
Venus mouse #3
Time from lick onset (s)
d
j
p
n = 7 mice
n = 6 mice
n = 6 mice
e
k
q
Mean ΔF/F0
Venus
axon-GCaMP
5-HT MRN→IPN
5-HT MRN→vHP
5-HT MRN→MHb
f
l
r
Distance (m/900 s)
Venus
eArchT
s
LHb
axon-GCaMP
Venus
n = 6 mice
t
5-HT MRN→LHb
u
PVT
axon-GCaMP
Venus
n = 4 mice
n = 6 mice
v
5-HT MRN→PVT

**Supplementary Fig. 7 | Projection-specific responses of Venus-expressing MRN serotonergic neurons to rewarding stimuli and effects of projection-specific optogenetic manipulation of MRN serotonergic neurons on locomotor activity.**

**a, g, m**, Schematic representation of the experiment. **b, h, n**, Representative trace of Venus fluorescence changes in the IPN (**b**), vHP (**h**), and MHb (**n**). **c, i, o**, Top: heatmap of signals (red–blue, high–low). One licking bout per row. Bottom: averaged trace of Venus signals of one mouse in the IPN (**c**), vHP (**i**), and MHb (**o**). Lines and shaded areas indicate mean and s.e.m., respectively. $n$ = 10 bouts. **d, j, p**, Mean Venus signals before and after the lick onset for 7 (IPN) or 6 (vHP, MHb) mice in the IPN (**d**), vHP (**j**), and MHb (**p**). **e, k, q**, The mean $\Delta F/F_0$ of 3-s after the lick onset from the IPN (**e**), vHP (**k**), and MHb (**q**). (Venus vs axon-GCaMP) IPN: $t_{7.895}$ = 4.302, $^{**}P$ = 0.0027, $n$ = 7 mice per group; vHP: $t_{10}$ = 4.228, $^{**}P$ = 0.0018, $n$ = 6 mice per group; MHb: $t_{5.561}$ = 1.458, $P$ = 0.1990, $n$ = 6 mice per group. **f, l, r**, The traveled distance in posttest session of the CPP test. (Venus vs eArchT) IPN (**f**): $t_{16}$ = 1.865, $P$ = 0.0807, $n$ = 9 mice per group; vHP (**l**): $t_{10.75}$ = 1.517, $P$ = 0.1580, $n$ = 9 mice per group; MHb (**r**): $t_{10.58}$ = 1.840, $P$ = 0.0939, $n$ = 9 mice per group. **s**, **u**, Representative trace of fluorescence changes before, during, and after sucrose solution licking in the LHb (**s**) and PVT (**u**). Blue segments indicate a statistically significant decrease from the baseline ($P$ < 0.05; permutation test). **t**, **v**, The mean $\Delta F/F_0$ of 3-s after the lick onset from the LHb (**t**) and PVT (**v**). (Venus vs axon-GCaMP) LHb: $t_{6.348}$ = 1.935, $P$ = 0.0984, $n$ = 6 mice per group; PVT: $t_8$ = 4.732, $P$ = 0.6487, $n$ = 6 (Venus) and 4 (axon-GCaMP) mice. Two-tailed unpaired $t$-test was used for statistical analyses. Data are presented as mean ± s.e.m. Error bars indicate s.e.m. Source data are provided as a Source Data file.

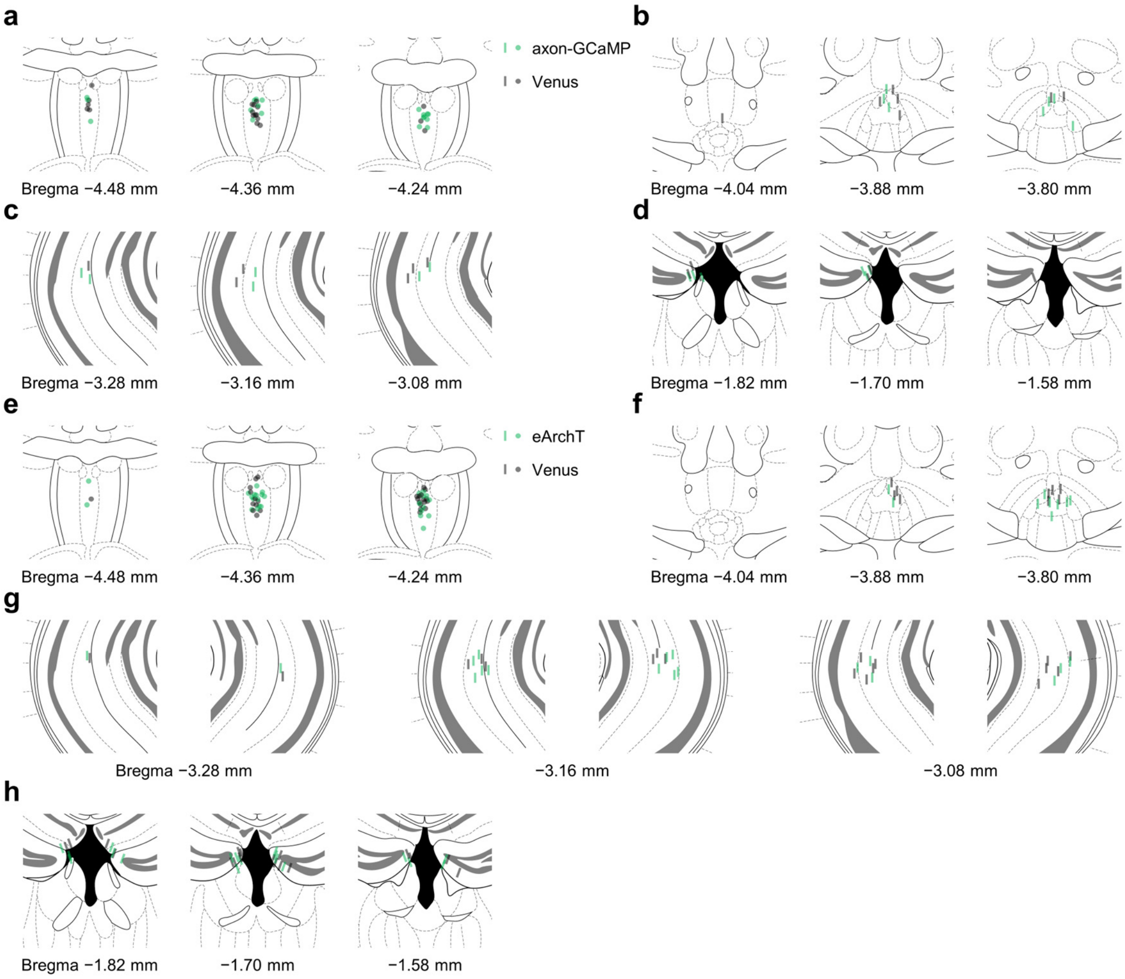


**Supplementary Fig. 8 | Verification of AAV injection and fiber implantation sites, related to Fig. 6.**

**a–d**, Verification of virus injection and fiber implantation sites in the MRN (**a**), IPN (**b**), vHP (**c**, unilaterally), and MHb (**d**) for fiber photometry recording during sucrose solution licking, related to Fig. 6. The AAV injection and fiber implantation sites were histologically verified under the microscope. Green circle: AAVdj-mTph2-axon-GCaMP6s-WPRE, Gray circle: AAVdj-mTph2-Venus-WPRE, Green line: the tip of fiber for axon-GCaMP mice, Gray line: the tip of fiber for Venus mice. **e–h**, Verification of virus injection and fiber implantation sites in the MRN (**e**), IPN (**f**), vHP (**g**, bilaterally), and MHb (**h**) for optogenetic manipulation in the CPP test, related to Fig. 6. The AAV injection and fiber implantation sites were histologically verified under the microscope. Green circle: AAVdj-mTph2-eArchT3.0-eYFP, Gray circle: AAVdj-mTph2-Venus-WPRE, Green line: the tip of the fiber for eArchT mice, Gray line: the tip of the fiber for Venus mice.

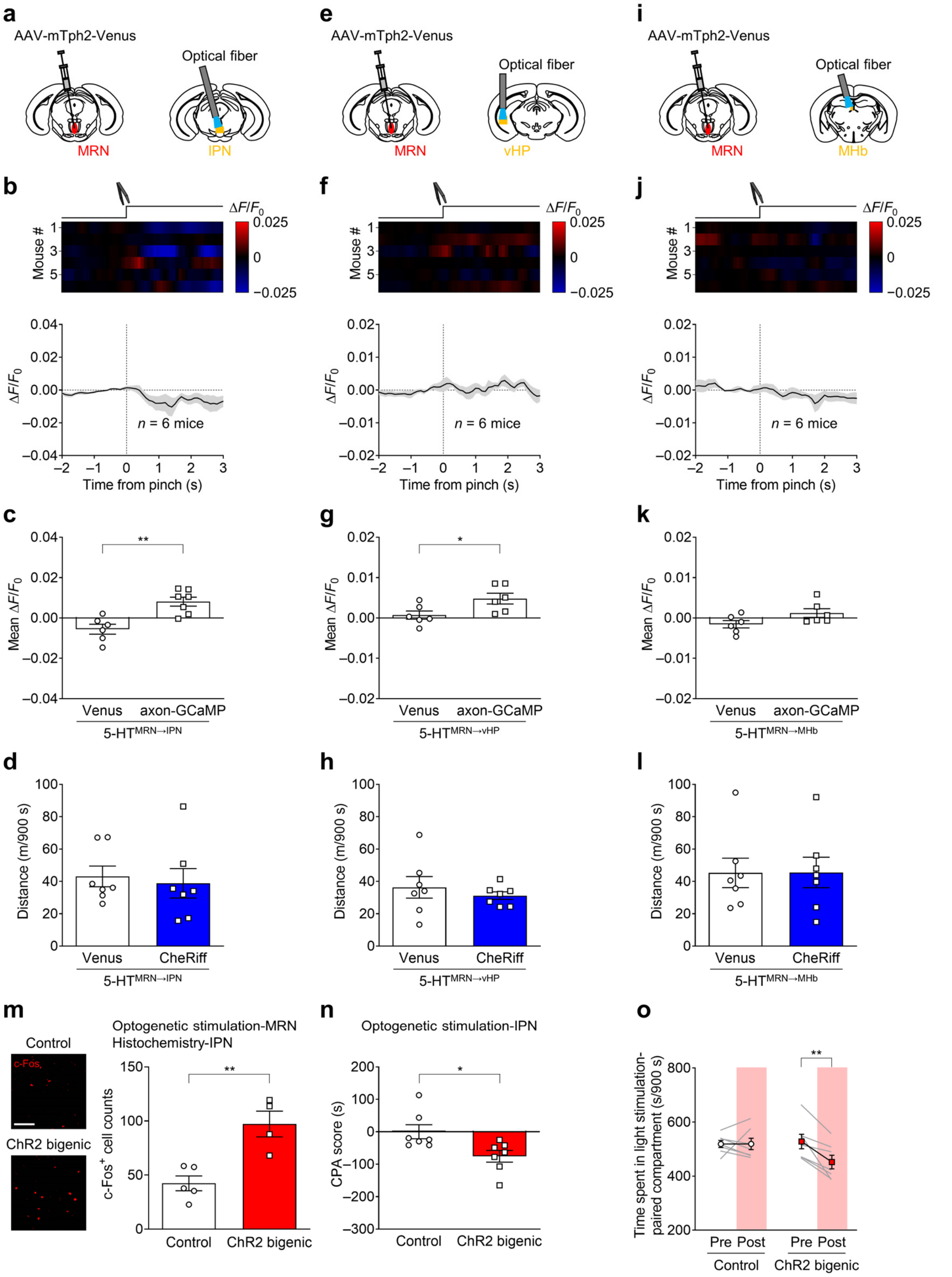


**Supplementary Fig. 9 | Projection-specific responses of Venus-expressing MRN serotonergic neurons to aversive stimuli, effects of projection-specific activation of MRN serotonergic neurons on locomotor activity, and**

**effect with optogenetic stimulation in ChR2(C128S) mice on c-Fos expression in the IPN and conditioned place aversion.**

**a, e, i**, Schematic representation of the experiment. **b, f, j**, Top: heatmap of signals (red–blue, high–low) for each trial (one trial per mouse). One mouse per row. $n$ = 6 mice. Bottom: averaged traces of Venus signals in the IPN (**b**), vHP (**f**), and MHb (**j**). Lines and shaded areas indicate mean and s.e.m., respectively. **c, g, k**, The mean $\Delta F/F_0$ of 3-s after pinch from the IPN (**c**), vHP (**g**), and MHb (**k**) for the axon-GCaMP mice and Venus control mice. (Venus vs axon-GCaMP) IPN: $t_{11} = 4.124$, $^{**}P = 0.0017$, $n$ = 6 (Venus) and 7 (axon-GCaMP) mice; vHP: $t_{10} = 2.437$, $^{*}P = 0.0350$, $n$ = 6 mice per group; MHb: $t_{10}$ = 1.962, $P = 0.0782$, $n$ = 6 mice per group. **d, h, l**, The traveled distance in posttest session of the CPA test. (Venus vs CheRiff) IPN (**d**): $t_{12} = 0.3838$, $P = 0.7079$, $n$ = 7 mice per group; vHP (**h**): $t_{7.460} = 0.7213$, $P = 0.4927$, $n$ = 7 mice per group; MHb (**l**): $t_{12} = 0.02146$, $P = 0.9832$, $n$ = 7 mice per group. **m**, 90-min after MRN stimulation, c-Fos expression in the IPN were analyzed. (Left) Representative images for c-Fos-positive (red) cells in the IPN. Scale bars = 50 µm. (Right) Quantification of c-Fos-positive cells in the IPN. $t_7$ = 4.21, $^{**}P = 0.004$, $n$ = 4 (ChR2 bigenic) and 5 (Control) mice. **n**, Activation of 5-HT terminals in the IPN of ChR2(C128S) mice promoted CPA. $t_{12} = 2.686$, $^{*}P = 0.0198$, $n$ = 7 mice. **o**, Spent time in the compartment associated with light stimulation in the IPN was significantly decreased after conditioning sessions (two-tailed paired $t$-test (Control), $t_6$ = 0.003358, $P = 0.9974$, $n$ = 7 mice; two-tailed paired $t$-test (ChR2 bigenic), $t_6 = 4.212$, $^{**}P$ = 0.0056, $n$ = 7 mice. Two-tailed unpaired $t$-test was used for statistical analyses unless otherwise stated. Data are presented as mean ± s.e.m. Error bars indicate s.e.m. Source data are provided as a Source Data file.

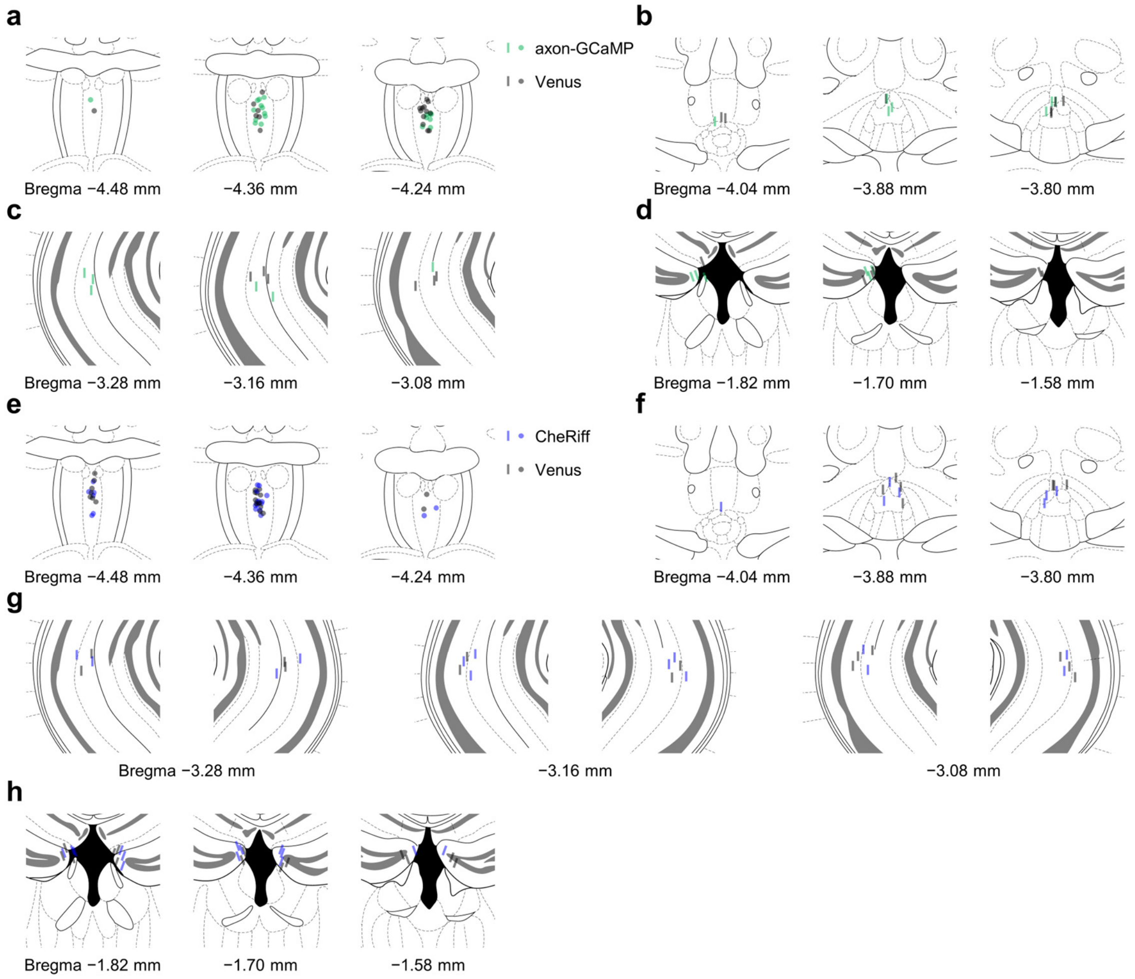


**Supplementary Fig. 10 | Verification of AAV injection and fiber implantation sites, related to Fig. 7.**

**a–d**, Verification of virus injection and fiber implantation sites in the MRN (**a**), IPN (**b**), vHP (**c**, unilaterally), and MHb (**d**) for fiber photometry recording during the tail pinch test, related to Fig. 7. The AAV injection site and the fiber implantation site were histologically verified under the microscope. Green circle: AAVdj-mTph2-axon-GCaMP6s-WPRE, Gray circle: AAVdj-mTph2-Venus-WPRE, Green line: the tip of fiber for axon-GCaMP mice, Gray line: the tip of fiber for Venus mice. **e–h**, Verification of virus injection and fiber implantation sites in the MRN (**e**), IPN (**f**), vHP (**g**, bilaterally), and MHb (**h**) for optogenetic manipulation of the CPA test, related to Fig. 7. AAV injection and fiber implantation sites were histologically verified under the microscope. Blue circle: AAVdj-mTph2-CheRiff-eGFP, Gray circle: AAVdj-mTph2-Venus-WPRE, Blue line: the tip of the fiber for CheRiff mice, Gray line: the tip of the fiber for Venus mice.

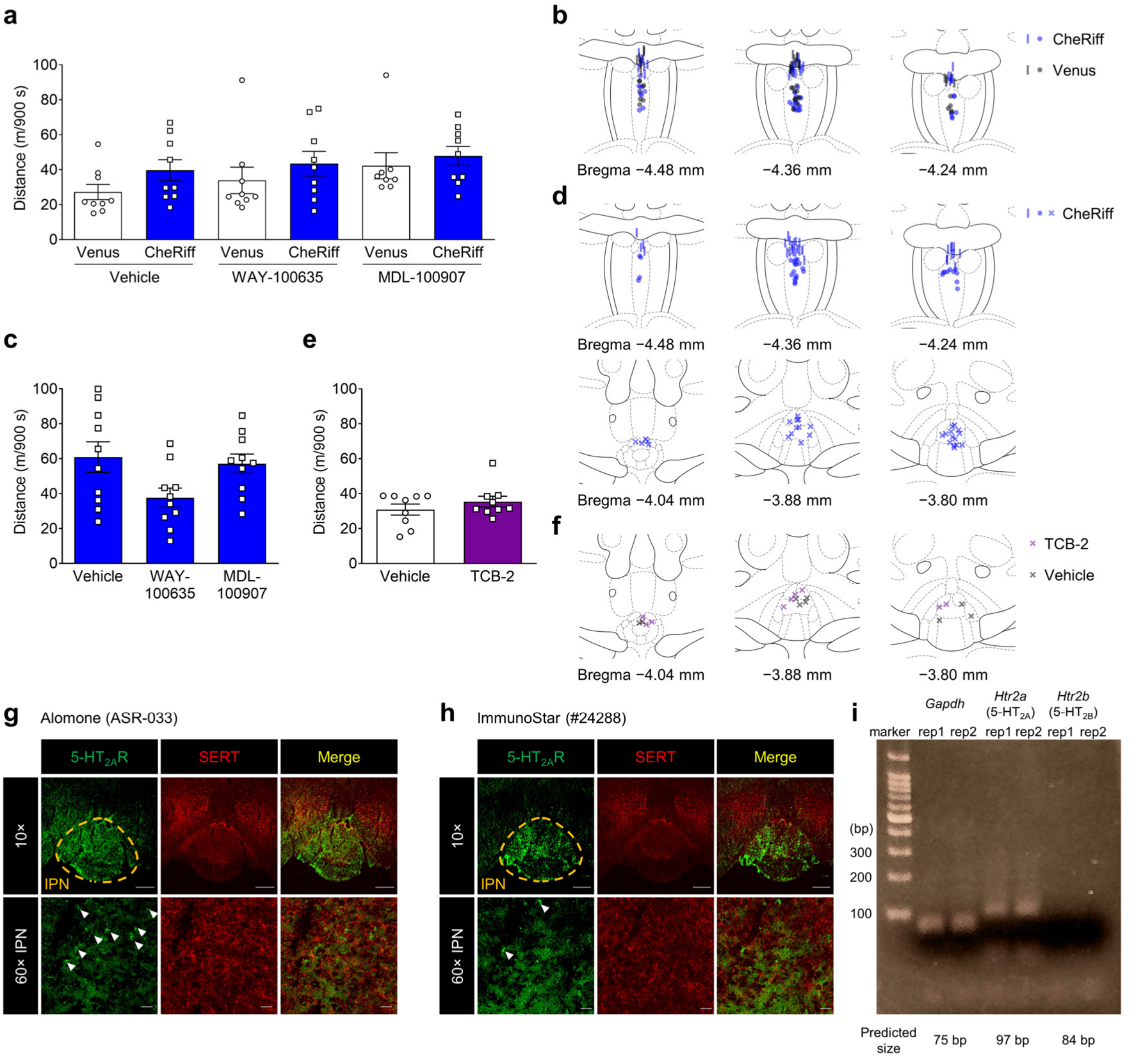


**Supplementary Fig. 11 | Effects of optogenetic activation of MRN serotonergic neurons with drug administration on locomotor activity and histochemical and genetic analyses of 5-HT2A receptor expression in the IPN.**

**a**, Traveled distance in posttest session of the CPA test. Two-way ANOVA with Tukey *post hoc* test, virus × drug interaction $F_{2,47} = 0.1441$, $P = 0.8662$, effect of virus $F_{1,47} = 3.119$, $P = 0.0839$, effect of drug $F_{2,47} = 1.638$, $P = 0.2052$, $n = 9$ (Venus-Vehicle, CheRiff-Vehicle, Venus-WAY-100635, CheRiff-WAY-100635 and CheRiff-MDL-100907) and 8 (Venus-MDL-100907) mice. **b**, Verification of virus injection and fiber implantation sites. Blue: CheRiff (circle), fiber tip (line), Gray: Venus (circle), fiber tip (line). **c**, Traveled distance in posttest session of the CPA test. One-way ANOVA with Tukey *post hoc* test, $F_{2,27} = 3.402$, $P = 0.0481$; *post hoc* test: Vehicle vs WAY-100635 $P = 0.0553$, Vehicle vs MDL-100907 $P = 0.9160$, WAY-100635 vs MDL-100907 $P = 0.1238$. $n = 10$ mice per

group. **d**, Verification of virus injection, fiber implantation, and cannula implantation sites in the MRN (top) and IPN (bottom). Blue circle: CheRiff, Blue line: fiber tip, Blue cross: cannula tip. **e**, Traveled distance in posttest session of the CPA test (two-tailed unpaired *t*-test (vehicle vs TCB-2), $t_{16}$ = 1.033, $P$ = 0.3168, $n$ = 9 mice per group). **f**, Verification of cannula implantation sites in the IPN in the CPA test with intra-IPN drug administration. Cannula implantation sites were verified under the microscope. Purple cross: the tip of the cannula for TCB-2-treated mice, Gray cross: the tip of the cannula for vehicle-treated mice. **g, h**, Coronal section containing IPN was stained with two antibodies for 5-$HT_{2A}$ receptor and anti-SERT antibody. Immunoreactivity of cell-like morphology was observed in the IPN. Scale bars = 200 μm (top) and 20 μm (bottom). **i**, RT-PCR analysis of cDNA of mouse IPN. PCR-amplified products were electrophoresed and visualized under UV. Data are presented as mean ± s.e.m. Error bars indicate s.e.m. Source data are provided as a Source Data file.

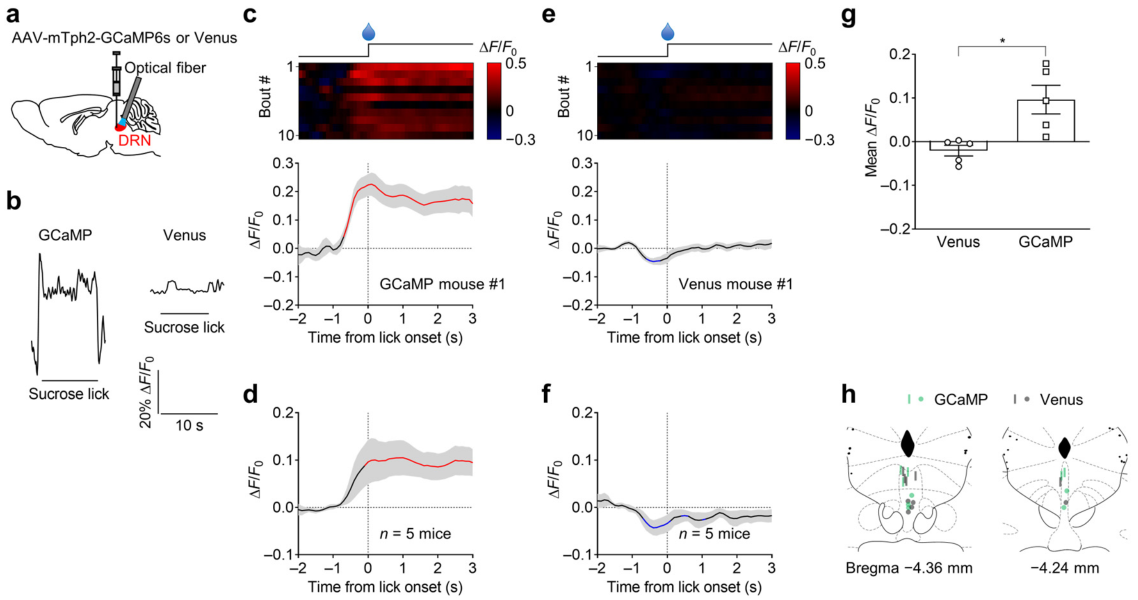


**Supplementary Fig. 12 | DRN serotonergic neurons are activated by rewarding stimuli.**

**a**, Schematic representation of AAV injection and fiber implantation sites. **b**, Representative raw traces of GCaMP (left) and Venus (right) fluorescence changes before, during, and after sucrose licking. **c, e**, Top: heatmaps of GCaMP (**c**) and Venus (**e**) signals (red–blue, high–low). Each row represents data from one licking bout. Bottom: averaged traces of GCaMP (**c**) and Venus (**e**) signals from the DRN in one behavioral session of one mouse. Lines and shaded areas indicate mean and s.e.m., respectively. Red and blue segments indicate statistically significant increase and decrease from the baseline, respectively ($P < 0.05$; permutation test). $n = 10$ bouts. **d, f**, The mean GCaMP (**d**) and Venus (**f**) signals before and after the lick onset for five mice. Red and blue segments indicate statistically significant increase and decrease from the baseline, respectively ($P < 0.05$; permutation test). **g**, The mean $\Delta F/F_0$ of 3-s after the lick onset in the GCaMP mice and Venus control mice (two-tailed unpaired *t*-test (Venus vs GCaMP), $t_8 = 3.333$, $^*P = 0.0103$, $n = 5$ mice per group). **h**, Verification of virus injection and fiber implantation sites in the DRN for fiber photometry recording during sucrose solution licking. AAV injection and fiber implantation sites were histologically verified under the microscope. Green circle: AAVdj-mTph2-GCaMP6s-WPRE, Gray circle: AAVdj-mTph2-Venus-WPRE, Green line: the tip of the fiber for GCaMP mice, Gray line: the tip of the fiber for Venus mice. Data are presented as mean ± s.e.m. Error bars indicate s.e.m.
