# Supplementary material for: Median raphe serotonergic neurons projecting to the interpeduncular nucleus control preference and aversion
Source: Nat Commun. 2022 Dec 22;13:7708. doi: 10.1038/s41467-022-35346-7 (PMC9780347; doi:10.1038/s41467-022-35346-7)
Supplement: Supplementary file 3 — Reporting Summary [file 41467_2022_35346_MOESM3_ESM.pdf]

nature portfolio

Corresponding author(s): Kazuki Nagayasu

Last updated by author(s): Nov 23, 2022

Reporting Summary

Nature Portfolio wishes to improve the reproducibility of the work that we publish. This form provides structure for consistency and transparency in reporting. For further information on Nature Portfolio policies, see our Editorial Policies and the Editorial Policy Checklist.

Statistics

For all statistical analyses, confirm that the following items are present in the figure legend, table legend, main text, or Methods section.

n/a	Confirmed	
☐	☒	The exact sample size (n) for each experimental group/condition, given as a discrete number and unit of measurement
☐	☒	A statement on whether measurements were taken from distinct samples or whether the same sample was measured repeatedly
☐	☒	The statistical test(s) used AND whether they are one- or two-sided<br>*Only common tests should be described solely by name; describe more complex techniques in the Methods section.*
☐	☒	A description of all covariates tested
☐	☒	A description of any assumptions or corrections, such as tests of normality and adjustment for multiple comparisons
☐	☒	A full description of the statistical parameters including central tendency (e.g. means) or other basic estimates (e.g. regression coefficient) AND variation (e.g. standard deviation) or associated estimates of uncertainty (e.g. confidence intervals)
☐	☒	For null hypothesis testing, the test statistic (e.g. F, t, r) with confidence intervals, effect sizes, degrees of freedom and P value noted<br>*Give P values as exact values whenever suitable.*
☒	☐	For Bayesian analysis, information on the choice of priors and Markov chain Monte Carlo settings
☒	☐	For hierarchical and complex designs, identification of the appropriate level for tests and full reporting of outcomes
☒	☐	Estimates of effect sizes (e.g. Cohen's d, Pearson's r), indicating how they were calculated

Our web collection on statistics for biologists contains articles on many of the points above.

Software and code

Policy information about availability of computer code

Data collection	ImageJ software plugin Micro-Manager (Edelstein, A. D. et al. Advanced methods of microscope control using μManager software. J. Biol. Methods 1, e10 (2014). https://micro-manager.org/)<br>A custom-written python script (https://github.com/YuYuB/MRN-Head-Fixed).<br>pClamp version 10.7 (Molecular Devices).
Data analysis	The facial expression analysis code (available on Github (https://github.com/GogollaLab) and at Dolensek, N. Gogolla, N. GogollaLab/ MouseFacialExpressionAnalysis, version 1.0, Zenodo (2020).)<br>R (version 4.0.3)<br>GraphPad Prism (version 8 and 9, GraphPad Software)<br>SPSS (version 23.0)

For manuscripts utilizing custom algorithms or software that are central to the research but not yet described in published literature, software must be made available to editors and reviewers. We strongly encourage code deposition in a community repository (e.g. GitHub). See the Nature Portfolio guidelines for submitting code & software for further information.

Data

Policy information about availability of data

All manuscripts must include a data availability statement. This statement should provide the following information, where applicable:

- Accession codes, unique identifiers, or web links for publicly available datasets
- A description of any restrictions on data availability
- For clinical datasets or third party data, please ensure that the statement adheres to our policy

The source data are provided as a Source Data file. Other data that support the findings of this study are available from the corresponding authors upon request.

Field-specific reporting

Please select the one below that is the best fit for your research. If you are not sure, read the appropriate sections before making your selection.

- [x] Life sciences
- [ ] Behavioural & social sciences
- [ ] Ecological, evolutionary & environmental sciences

For a reference copy of the document with all sections, see nature.com/documents/nr-reporting-summary-flat.pdf

Life sciences study design

All studies must disclose on these points even when the disclosure is negative.

Sample size	The sample size was determined based on similar studies in this field. The list of references is as follows: Li, Y. et al. Serotonin neurons in the dorsal raphe nucleus encode reward signals. Nat. Commun. 7, 10503 (2016). Broussard, G. J. et al. In vivo measurement of afferent activity with axon-specific calcium imaging. Nat. Neurosci. 21, 1272–1280 (2018). Lammel, S. et al. Input-specific control of reward and aversion in the ventral tegmental area. Nature 491, 212–217 (2012). Yang, H. et al. Nucleus accumbens subnuclei regulate motivated behavior via direct inhibition and disinhibition of VTA dopamine subpopulations. Neuron 97, 434–449.e4 (2018). Yang, H. et al. Pain modulates dopamine neurons via a spinal–parabrachial–mesencephalic circuit. Nat. Neurosci. 24, 1402–1413 (2021).
Data exclusions	No data were excluded from the analysis, except for the mice with failed AAV infection, fiber implantation, or cannula implantation.
Replication	The experiments were performed independently. Pooled results were analyzed in total and shown in the manuscript.
Randomization	Mice were randomly assigned to each experimental group.
Blinding	Blinding was not formally performed because all experiments were automatically analyzed by software which is naturally unaware of experimental groups.

Reporting for specific materials, systems and methods

We require information from authors about some types of materials, experimental systems and methods used in many studies. Here, indicate whether each material, system or method listed is relevant to your study. If you are not sure if a list item applies to your research, read the appropriate section before selecting a response.

Materials & experimental systems

n/a	Involved in the study	
[ ]	[x]	Antibodies
[ ]	[x]	Eukaryotic cell lines
[x]	[ ]	Palaeontology and archaeology
[ ]	[x]	Animals and other organisms
[x]	[ ]	Human research participants
[x]	[ ]	Clinical data
[x]	[ ]	Dual use research of concern

Methods

n/a	Involved in the study	
[x]	[ ]	ChIP-seq
[x]	[ ]	Flow cytometry
[x]	[ ]	MRI-based neuroimaging

Antibodies

Antibodies used

1. rabbit polyclonal anti-green fluorescent protein antibody (Thermo Fisher Scientific, A-11122, lot: 1891900, 2083201, 2180255, 2273763)
2. sheep polyclonal anti-tryptophan hydroxylase antibody (Merck Millipore, AB1541, lot: 2809957, 316027, 3442376)
3. goat polyclonal anti-serotonin transporter antibody (Nittobo Medical, HTT-Go-Af970, lot: AF970)
4. mouse monoclonal anti-c-Fos antibody (2H2) (Novus Biologicals, NBP2-50037, clone name: 2H2, lot: 021420)
5. mouse monoclonal anti-green fluorescent protein antibody (mFX73) (WAKO, 012-20461, clone name: mFX73, lot: LKN5942)
6. rabbit anti-tryptophan hydroxylase 2 (Nakamura, K., Sato, T., Ohashi, A., Tsurui, H. & Hasegawa, H. Role of a serotonin precursor in

development of gut microvilli. Am. J. Pathol. 172, 333–344 (2008).)
7. Alexa Fluor 594-labeled donkey anti-rabbit IgG (Thermo Fisher Scientific, A-21207, lot: 1827074, 1938375, 1987293, 2066086, 2155297, 2266563)
8. Alexa Fluor 647-labeled donkey anti-sheep IgG (Thermo Fisher Scientific, A-21448, lot: 1774718, 2045339)
9. Alexa Fluor 488-labeled donkey anti-rabbit IgG (Thermo Fisher Scientific, A-21206, lot: 2289872)
10. Alexa Fluor 594-labeled donkey anti-goat IgG (Thermo Fisher Scientific, A-11058, lot: 2185074)
11. Alexa Fluor 594-labeled donkey anti-sheep IgG (Thermo Fisher Scientific, A-11016, lot: 2044344, 2155297)
12. Alexa Fluor 647-labeled donkey anti-mouse IgG (Thermo Fisher Scientific, A-31571, lot: 2136787)
13. Alexa Fluor 488-labeled donkey anti-mouse IgG (Invitrogen, A-21202, lot: 1975519)
14. Cy3 AffiniPure donkey anti-rabbit IgG (Jackson ImmunoResearch, 711-165-152, lot: 141941)
15. rabbit anti-5-HT2A receptor antibody (Alomone, ASR-033)
16. rabbit anti-5-HT2A receptor antibody (Immunostar, 24288, lot: 2041001L)

Validation

1. https://www.thermofisher.com/antibody/product/GFP-Antibody-Polyclonal/A-11122
2. https://www.merckmillipore.com/GB/en/product/Anti-Tryptophan-Hydroxylase-Antibody,MM_NF-AB1541
3. https://nittobo-nmd.co.jp/pdf/reagents/HTT.pdf
4. https://www.novusbio.com/products/c-fos-antibody-2h2_nbp2-50037
5. https://labchem-wako.fujifilm.com/europe/product/detail/W01W0101-2254.html
6. Nakamura, K., Sato, T., Ohashi, A., Tsurui, H. & Hasegawa, H. Role of a serotonin precursor in development of gut microvilli. Am. J. Pathol. 172, 333–344 (2008).
7. https://www.thermofisher.com/antibody/product/Donkey-anti-Rabbit-IgG-H-L-Highly-Cross-Adsorbed-Secondary-Antibody-Polyclonal/A-21207
8. https://www.thermofisher.com/antibody/product/Donkey-anti-Sheep-IgG-H-L-Cross-Adsorbed-Secondary-Antibody-Polyclonal/A-21448
9. https://www.thermofisher.com/antibody/product/Donkey-anti-Rabbit-IgG-H-L-Highly-Cross-Adsorbed-Secondary-Antibody-Polyclonal/A-21206
10. https://www.thermofisher.com/antibody/product/Donkey-anti-Goat-IgG-H-L-Cross-Adsorbed-Secondary-Antibody-Polyclonal/A-11058
11. https://www.thermofisher.com/antibody/product/Donkey-anti-Sheep-IgG-H-L-Cross-Adsorbed-Secondary-Antibody-Polyclonal/A-11016
12. https://www.thermofisher.com/antibody/product/Donkey-anti-Mouse-IgG-H-L-Highly-Cross-Adsorbed-Secondary-Antibody-Polyclonal/A-31571
13. https://www.thermofisher.com/antibody/product/Donkey-anti-Mouse-IgG-H-L-Highly-Cross-Adsorbed-Secondary-Antibody-Polyclonal/A-21202
14. https://www.jacksonimmuno.com/catalog/products/711-165-152
15. https://www.alomone.com/p/anti-5-hydroxytryptamine-receptor-2a-extracellular/ASR-033?gclid=EAIaIQobChMIpeWqgPHj-QIVj66WCh2N4wc-EAAYASAAEgLqafD_BwE
16. https://www.immunostar.com/product/5-ht-serotonin-2a-receptor-antibody/

Eukaryotic cell lines

Policy information about cell lines

Cell line source(s)	Lenti-X 293T cells (human, clontech, 632180)
Authentication	None of the cell lines have been authenticated.
Mycoplasma contamination	Cell lines were not tested for mycoplasma contamination, but no indication of contamination was observed.
Commonly misidentified lines (See ICLAC register)	No commonly misidentified cell lines were used.

Animals and other organisms

Policy information about studies involving animals; ARRIVE guidelines recommended for reporting animal research

Laboratory animals

C57BL/6J mice (JmsSlc, male, 7–11 weeks old; Japan SLC)
C57BL/6N mice (JmsSlc, male, aged > 70 days; Japan SLC)
Tph2-tTA::tetO-ChR2(C128S)-eYFP bi-transgenic mice (male and female, aged 8-20 weeks old). Approximately 50% of the bi-transgenic mice were female. tetO-ChR2(C128S)-eYFP knock-in mice (RRID:IMSR_RBRC05454) and Tph2-tTA BAC transgenic mice (RRID:IMSR_RBRC05846) were used only for the production of bi-transgenic mice and not used for experiments. These mice have been described in previous reports (Tanaka, K. F. et al. Expanding the repertoire of optogenetically targeted cells with an enhanced gene expression system. Cell Rep. 2, 397–406 (2012)., and Ohmura, Y., Tanaka, K. F., Tsunematsu, T., Yamanaka, A. & Yoshioka, M. Optogenetic activation of serotonergic neurons enhances anxiety-like behaviour in mice. Int. J. Neuropsychopharmacol. 17, 1777–1783 (2014).).

Wild animals

No wild animals were used in this study.

Field-collected samples

No field-collected samples were used in this study.

Ethics oversight

The mouse work was performed under the ethical guidelines of the Kyoto University animal research committee (approval code: 13-41-2, 19-41-1,2,3), the animal research committee of Hokkaido University (approval code: 18-0070), and the animal research

committee of Kanazawa University (approval code: AP-204167). , as approved by the Institutional Animal Care and Use Committee.

Note that full information on the approval of the study protocol must also be provided in the manuscript.
